# Pharmacological Activities and Mechanisms of Hirudin and Its Derivatives - A Review

**DOI:** 10.3389/fphar.2021.660757

**Published:** 2021-04-16

**Authors:** Chen Junren, Xie Xiaofang, Zhang Huiqiong, Li Gangmin, Yin Yanpeng, Cao Xiaoyu, Gao Yuqing, Li Yanan, Zhang Yue, Peng Fu, Peng Cheng

**Affiliations:** ^1^ State Key Laboratory of Southwestern Chinese Medicine Resources, Chengdu, China; ^2^ Chengdu University of Traditional Chinese Medicine, Chengdu, China; ^3^ West China School of Pharmacy, Sichuan University, Chengdu, China

**Keywords:** natural hirudin, recombinant hirudin, anti-thrombosis, wound repair, anti-fibrosis, mechanisms

## Abstract

Hirudin, an acidic polypeptide secreted by the salivary glands of *Hirudo medicinalis* (also known as “Shuizhi” in traditional Chinese medicine), is the strongest natural specific inhibitor of thrombin found so far. Hirudin has been demonstrated to possess potent anti-thrombotic effect in previous studies. Recently, increasing researches have focused on the anti-thrombotic activity of the derivatives of hirudin, mainly because these derivatives have stronger antithrombotic activity and lower bleeding risk. Additionally, various bioactivities of hirudin have been reported as well, including wound repair effect, anti-fibrosis effect, effect on diabetic complications, anti-tumor effect, anti-hyperuricemia effect, effect on cerebral hemorrhage, and others. Therefore, by collecting and summarizing publications from the recent two decades, the pharmacological activities, pharmacokinetics, novel preparations and derivatives, as well as toxicity of hirudin were systematically reviewed in this paper. In addition, the clinical application, the underlying mechanisms of pharmacological effects, the dose-effect relationship, and the development potential in new drug research of hirudin were discussed on the purpose of providing new ideas for application of hirudin in treating related diseases.

## Introduction

**FIGURE 1:**
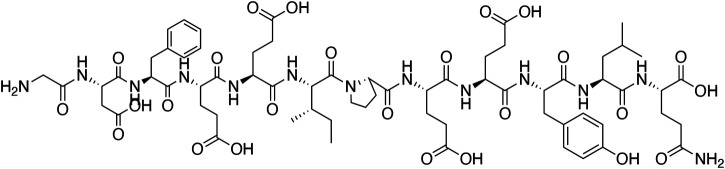
The chemical structure of natural hirudin.

*Hirudo medicinalis*, belonging to hematophagous leeches, has been used for medical purposes since ancient times. Originally recorded in *Shennong's Classic of Material Medical*, leeches have the efficacy of breaking blood, expelling stasis and freeing the collateral vessels in traditional Chinese medicine (TCM) (Chinese Pharmacopoeia Committee, 2020). Natural hirudin is a polypeptide isolated from the salivary glands of the *Hirudo medicinalis*, which is composed of 64–66 amino acids with a molecular weight of about 7000 Da. Hirudin was firstly found as early as in 1884, when Haycraft found that the extract from *Hirudo medicinalis* took on anticoagulant property, and later the extract was named hirudin by Jacoby in 1904 (Mammen, 1983; Markwardt, 1991b). In 1955, Markward successfully obtained the relatively pure hirudin from the salivary glands of *Hirudo medicinalis*, which greatly facilitated the research of thrombin inhibitors (Fields, 1991). In 1984, Dodt *et al.* firstly described the complete amino acid sequence of hirudin (Dodt et al., 1984). Subsequently, several subtypes of hirudin were discovered, such as hirudin variant 1 (HV1, or hirudin- VV), hirudin variant 2 (HV2, or hirudin-IT), and hirudin variant 3 (HV3, or hirudin-PA), all of which have highly similar core sequences and are highly specific thrombin inhibitors (Johnson et al., 1989). From then on, numerous variants of the three subtypes of hirudin were described (Scharf et al., 1989), and more than ten diverse hirudin variants (HVs) have been discovered in leech so far. These peptides possess high stability and activity, with the minimum dissociation constant of 2.3 × 10^–14^ mol, and the maximum association constant for binding to thrombin of 4.7 × 108 mol^−1^ S^−1^ (Zhang and Lan, 2018). Notably, all HVs have six Cys residues, and anticoagulant peptides without these residues are not considered members of the hirudin family (Strube et al., 1993). The chemical structure of natural hirudin is shown in Figure 1.

Hirudin is an effective natural thrombin inhibitor, however, the low production of natural hirudin limits its research and application. With the development of bioengineering technology, recombinant hirudin (rH) was synthesized by genetic engineering, which possesses similar chemical structure and pharmacological activity to natural hirudin. In 1986, Bergmann *et al.* obtained rH in *Escherichia coli* using chemically synthesized hirudin genes, which possesses similar anti-thrombotic property as the natural compound (Bergmann et al., 1986). In 1988, Loison *et al.* used *Saccharomyces. cerevisiae* to get the efficient production of rHV2 with the expression level of 21 ATU/ml (Loison et al., 1988). Furthermore, Sohn *et al.* optimized the expression vector and fermentation conditions, enabling the expression level of recombinant hirudin to reach to 460 mg/L (Sohn et al., 1995). In 1996, Rosenfeld *et al.* synthetized rH in *P. pastoris*, and the expression level reached 1.5g/L (Rosenfeld et al., 1996). In recent decades, the hosts for expressing rH are also increasing, which vary from *Bacillus subtilis* (Chen et al., 2011) to *Lactococcus lactis* (Lv et al., 2012). rH can be produced not only by bacterial or yeast expression systems, but also by a cell-free protein synthesis approach, which can express rH with higher antithrombin activity (Wustenhagen et al., 2020). Although these methods can increase the expression of recombinant hirudin, the thrombin inhibition constant is still lower than that of natural hirudin, mainly because it lacks the tyrosine residue sulfated at site 63 (Nowak and Schror, 2007). Recently, in order to improve the bioavailability and reduce the adverse reactions of hirudin, more and more hirudin derivatives have been constantly synthesized and developed, such as recombinant-RGD-hirudin, neorudin, Annexin V-hirudin 3-ABD, Boronophenylalanine-modified hirudin, and rhSOD2-hirudin. Moreover, several derivatives like lepirudin, desirudin, and bivalirudin have been applied in clinic for thrombotic diseases.

In recent years, numerous studies of hirudin and its derivatives have rapidly risen, especially on effects of wound repair, anti-fibrosis, and anti-diabetic complications effects. In addition, the studies of other pharmacological effects including anti-thrombotic effect, anti-tumor effect, anti-hyperuricemia effect, and effects on cerebral hemorrhage, IgA nephropathy, acute lung injury, as well as myocardial infraction have also made some progress. The underlying molecular mechanisms of these pharmacological properties have been partly revealed. Nowadays, to improve some specific pharmacological activities and decrease the adverse effects of hirudin, scientists have been devoted to synthetizing derivatives and innovating preparations of hirudin, and gained some exciting findings. This review will provide detailed active effects of hirudin and its derivatives, as well as the toxicity.

## Pharmacological Effects

### Anti-thrombotic Effect

#### Natural Hirudin

As a potent antithrombotic peptide extracted from leeches, hirudin has become the most specific tight-binding thrombin inhibitor since it was isolated in the late 1950s (Matheson and Goa, 2000). The highly specific inhibitory effect of hirudin on thrombin is closely related to its chemical structure. Three pairs of disulfide bonds have been proved in the N-end of hirudin (Cys6-Cys14, Cys16-Cys28, Cys32-Cys39), which form a compact structure that can bind to the active site of thrombin. Besides, the C-end is rich in acidic amino acid residues, which can bind to the fibrinogen binding site of thrombin, thus inhibiting the anticoagulation of thrombin (Nowak, 2002). In addition, a special sequence composed of Pro-Lys-Pro has been described in the middle of the peptide chain, which can not only maintain the stability of hirudin, but also guide the precise binding of hirudin and thrombin (Markwardt, 1994).

Thrombin exerts a significant role in the clotting system, it converts fibrinogen to fibrin, and facilitates the stabilization of fibrin and the amplification of fibrin formation by activating factor XIII, V as well as VIII. Besides, it is also a potent activator of platelets, which can contribute to platelet-fibrin thrombosis. Noticeably, hirudin was reported to possess inhibitory effect in these processes (Markwardt, 1991a; Monreal et al., 1996). Different from other anticoagulant drugs such as heparin and sodium citrate, hirudin inhibits the activity of thrombin without the cofactor antithrombin III (Dodt et al., 1985) and combines with thrombin at the molar ratio of 1 ∶1. Besides, hirudin deprives the ability of thrombin to cleave fibrinogen, which prevents the formation of fibrin and the cross-linking polymerization process of fibrin monomer in internal and external coagulation pathway.

Given the potent antithrombotic property, hirudin has been used to treat acute coronary artery disease, deep vein thrombosis and other thrombotic diseases (Zoldhelyi et al., 1993; Levin and Bergqvist, 2001). However, due to the disadvantages such as high cost, short half-life and easy to cause bleeding, the clinical application of natural hirudin is limited. At present, the structural modification of hirudin and the targeted drug delivery systems have attracted the attention of researchers, which were benefit to solve the problems mentioned above.

#### The Derivatives of Hirudin

After decades of development, several derivatives have been exploited and successfully applied in clinic for thrombotic diseases treatment, such as lepirudin, desirudin, and bivalirudin. Lepirudin is a 65-peptide rH with a molecular weight of 6979.5 KDa, which can directly inhibit the active site pocket and the fibrinogen binding site of free and clot-bound thrombin (Greinacher and Lubenow, 2001). Desirudin is also a direct thrombin inhibitor, which possesses a similar amino acid sequence of lepirudin, with the only difference lying on the first to second amino acids at the amino terminal (Lee and Ansell, 2011). The amino acid residue sequence of lepirudin at that site is Lue1-Thr2, while that of desirudin is Val1-Val2. Additionally, as an analogue of hirudin, bivalirudin is a synthetic polypeptide with the molecular weight of 2180, which can inhibit platelet aggregation but do not increase platelet reactivity. Dramatically, the binding between bivalirudin and thrombin is reversible, mainly because of the peptide bond between Arg3-Pro4 can be easily broken by thrombin (Scatena, 2000).

In recent years, a number of novel derivatives have been exploited because of the great demand for hirudin in physicochemical and clinical studies, including recombinant RGD-hirudin, boronophenylalanine-modified hirudin, neorudin, Annexin V-hirudin 3-ABD, and others. From these studies, it can be concluded that advantages of these derivatives of hirudin include reducing the bleeding risks through targeting thrombus sites and increasing the antithrombotic efficacy. The details of these derivatives are shown in Table 1.

**TABLE 1:** The Specialties of hirudin and its derivatives.

Derivatives	Effective constituent	Hosts	Specialties	References
Hirudin	-	*Hirudo medicinalis*	Exhibiting stronger antithrombotic effect compared with rH; possessing lower side effects of hemorrhage, nonallergic reaction, and non-toxicity compared with heparin; irreversibly binding with thrombin	Markwardt (1994)
			Short half-life; exhibiting bleeding risk after repeated injections	
Lepirudin	rH	*S. cerevisiae*	Leu^1^-thr^2^-desulfato-hirudin (65-amino acid polypeptide); irreversibly binding with thrombin; antithrombin activity: 18,000 ATU/mg	Greinacher and Lubenow (2001)
Desirudin	rH	*S. cerevisiae*	Desulfato-hirudin (65-amino-acid polypeptide); irreversibly binding with thrombin; antithrombin activity: 16,000 ATU/mg	Matheson and Goa (2000)
Bivalirudin	Hirulog	-	C-terminal hirudin region linked by polyglycine spacer to tetrapeptide reactive with thrombin active site (20-amino-acid polypeptide); reversibly binding with thrombin	Warkentin et al. (2008)
EPR-hirudin	Hirudin	*Pichia pastoris*	Exhibiting obvious antithrombotic effects and lower bleeding parameters than hirudin *in vivo*	Zhang et al. (2008)
GVYAR-hirudin				
LGPR-hirudin				
Recombinant-RGD-hirudin	Hirudin	*Pichia pastoris*	Exhibiting two to three times more effective than hirudin in preventing thrombosis and possessing the capacity to inhibit platelet aggregation	Mo et al. (2009)
EPR-HV3HSA	HV3	*Pichia pastoris*	Inhibiting thrombosis *in vivo* without promoting blood loss	Sheffield et al. (2018)
DTIP	RGD-hirudin	-	Exhibiting fewer bleeding risks than bivalirudin and exerting antithrombotic effects via subcutaneous injection	Zhao et al. (2017); Li et al. (2020)
Annexin V-hirudin 3-ABD	Hirudin	*E. coli*	Shortening bleeding time, prolonging circulation *in vivo*, and targeting coagulation site to exert antithrombin activity	Han H.-H. et al. (2020)
Neorudin	HV2-Lys47	*E. coli*	Maintaining the anti-thrombin activity of hirudin with fewer bleeding side effects risks	Dong et al. (2017)
HV12p-rPA	Hirudin-PA	*E. coli*	Exhibiting both fibrinolytic and anticoagulant activities	Gao et al. (2012)
rH-I analogue expressed with ASST	rH	*E. coli*	Exhibiting anticoagulant activity twice more than rH-I analogue without ASST and 4.5 times higher anticoagulant activity compared to heparin	Rai et al. (2020)

##### Decreasing the Bleeding Risk

Due to the risk of bleeding associated with hirudin, most of the researches on its derivatives have focused on reducing the risk of bleeding. Interestingly, the construction of new hirudin derivatives or fusion proteins that control the release of hirudin at specific sites has been proved as an effective way to reduce the side effects of hirudin.

In 2008, three derivatives of hirudin including EPR-hirudin, GVYAR-hirudin, and LGPR-hirudin were designed, in order to decrease the bleeding risk of hirudin. ERP, GVYAR, and LGPR belonging to the recognition peptides of factor XIa (FXIa), FXa, and thrombin, respectively, were linked to the N-terminus of hirudin in these derivatives. Zhang *et al.* reported that these derivatives exhibited significant antithrombotic effects in rats with lower bleeding risk than hirudin (Zhang et al., 2008). Neorudin, a novel anticoagulant fusion protein, was designed as a prodrug that releases hirudin variant 2-Lys47 (HV2) targeting the thrombosis site. Constructed by adding the amino acid sequence of EPR (Glu, Pro, and Arg) identified from the coagulation factors Xa and XIa into the N-terminus of hirudin, this fusion protein can release HV2 only at the thrombosis site to treat deep vein thromboembolism without systemic bleeding Wang et al., 2013; Dong et al., 2018). Recently, Sheffield *et al.* combined albumin fusion for half-life extension of hirudin with positioning of an FXIa cleavage site N-terminal to HV3 to assess the properties of this novel protein *in vitro* and *in vivo*. They discovered that FXIa-cleavable EPR-HV3HSA reduced the time to occlusion of ferric chloride-treated murine arteries and reduced fibrin deposition in murine endotoxemia, which implied that releasing the N-terminal block to HV3 activity using FXIa was an effective way to limit hirudin’s bleeding side-effects (Sheffield et al., 2018). Besides, a fusion protein Annexin V-hirudin 3-ABD (hAvHA) was constructed by Han *et al.* for prolonged circulation and targeted delivery of hirudin to coagulation sites, which could combine with albumin during circulation. When clotting occurs, the drug will be activated via FXa-mediated cleavage, which ensures the efficient release of hirudin and its antithrombin activity *in vitro*. In addition, hAvHA could prolong circulation *in vivo* by closely binding to human serum albumin. The tail bleeding assay in mice showed that the bleeding time was much shorter after the administration of hAvHA compared with hirudin (Han H.-H. et al., 2020).

##### Increasing the Antithrombotic Efficacy

Although the production of hirudin can be enhanced by bioengineering methods, the reduced effectiveness of rH remains a serious drawback, mainly due to the lack of sulfation of tyrosine 63 in rH (Liu et al., 2007). By means of molecular structural modification, rH showed the platelet aggregation inhibitory and fibrinolytic properties, which compensated that deficiency to a certain degree.

Recombinant-RGD-hirudin is a novel bi-functional hirudin molecule constructed by fusing the tripeptide Arg-Gly-Asp (RGD) sequence to the wild-type HV2, which could be expressed in *Pichia pastoris* at high levels. It can bind to glycoprotein IIb/IIIa of platelets to inhibit the aggregation of platelet. Remarkably, this new anti-coagulant showed two to three times more effective than wild-type hirudin in preventing thrombosis, with a much lower dosage (Mo et al., 2009). However, recombinant-RGD-hirudin can only be administered intravenously, which limited its clinical application (Zhao et al., 2017). Therefore, Li *et al.* designed a novel direct thrombin inhibitor peptide (DTIP) based on the structure and function of RGD-hirudin. It was shown that DTIP could significantly delay blood coagulation and prevent thrombosis via subcutaneous injection (Li et al., 2020). Besides, HV12p-rPA is a fusion protein composed of C-terminal 12-residue of hirudin-PA (HV12p) and reteplase (rPA), which combines the biological specialties of its components and thus possesses both fibrinolytic and anticoagulant properties. This bifunctional protein might be greater specificity for the thrombus, and has longer circulating time *in vivo* with low risk of hemorrhage due to its structural features, but further studies are needed to confirm these characters (Gao et al., 2012). Recent studies reported the co-expression of rH-I analogues with arylsulfotransferase (ASST) in *Escherichia coli* BL21 (DE3). Co-expressed rH-I analogue with sulfate donor substrate (p-nitrophenyl sulfate potassium) showed anticoagulant (rabbit and goat serum) activity twice more than rH-I analogue expressed without ASST. In addition, purified rH-I analogue showed above 4.5 times higher anticoagulant activity compared to therapeutic anti-thrombotic heparin (Rai et al., 2020). These research progress of hirudin in structure modification may greatly promote its clinical application and overcome its original defects.

#### Hirudin-like Factors

More recently, the hirudin-like factors (HLFs), possessing the unique structural characteristics of hirudin, are considered as the subclass of compounds derived from the salivary glands of *Hirudo medicinalis* and *Hirudinaria manillensis* (Muller et al., 2016). Muller *et al.* reported that a similar N-terminal structure which contains hydrophobic amino acid residues were reported in positions 1 and 3 of all HLFs, as well as a variable residue in position 2. In addition, they proposed that hirudins might be the particular HLFs targeted to thrombin according to the details from the conserved gene structure (Muller et al., 2017), thus, investigating the full genome data of these *Hirudo* species might be beneficial to reveal the relationships between function and phylogeny of leeches. So far, eight HLFs have been identified in leeches, including HLF1, HLF 2, HLF 3, HLF 4, HLF 5, HLF 6, HLF 8 and HLF-Hyb. Among them, only HLF5 and HLF8 exhibit anticoagulatory activities (Lukas et al., 2019), but the inhibitory activity of them was much lower than that of HV1 and HV2. Despite HLF1 has no antithrombotic activity in previous studies, Muller *et al.* found that similar thrombin-inhibiting activity to hirudins could be observed in HLF1 after N-terminal modification. Additionally, analogous phenomena could be found in HLF3 and HLF4 through modifications within the N-termini in combination with an exchange of the central globular domain (Muller et al., 2020) (Müller et al., 2020). These results suggested that the N- and C-termini as well as the central globular domains are crucial for hirudin and HLFs to exert antithrombotic activity.

## Wound Repair Effect

Random skin flap is common for repairing wound, reconstructing the function and improving skin appearance. However, necrosis of the skin flap is the biggest obstacle after flap transplantation, which is influenced by a diversity of factors, including flap venous congestion, blood circulation disorder, flap infection and flap avulsion (Yan et al., 2010). As a predominant regulator of angiogenesis, endogenous vascular endothelial growth factor (VEGF) plays important roles in flap survival that can promote endothelial cell proliferation and angiogenesis (Apte et al., 2019). In addition, VEGF can interconnect with multiple cellular pathways such as Notch, p38 MAPK, as well as ERK1/2 to exert the proangiogenic effect (Park et al., 2016; Chen J. et al., 2019), which indicated that regulating these pathways may improve the survival rate of random skin flaps. Remarkably, during the transfer process of random flap transplantation, an obligatory period of ischemia is required, which can initiate many inflammatory cytokines and produce damage in tissues (Peng et al., 2015). Therefore, inhibiting the development of inflammatory response is also essential for random flap transplantation. Even though many studies have been implemented to improve the blood circulation, promote angiogenesis, and alleviate inflammation of the flap, there is no good recognized method to improve flap survival. Multiple studies have proved that hirudin could exert outstanding therapeutic effect in treating ischemic flaps by increasing the surviving area, improving the microcirculation, reducing tissue inflammation, and promoting blood vessel regeneration as well as the recanalization of ischemic flaps (Zhao et al., 2012; Yin et al., 2014; Yan et al., 2020). Part of the potential signaling pathways have been elucidated including p38-MAPK/NF-**κ**B, JAK2/STATs, VEGF/Notch, VEGF/p38 MAPK/ERK1/2, and MCP-1/NF-κB, while some of the molecular mechanisms associated with oxidative stress need to be further investigated.

### (PARs)/p38/NF-**κ**B

Proteinase-activated receptors (PARs), a family of G protein–coupled receptors (GPCRs), are expressed in many cell types in the vasculature, such as platelets, smooth muscle cells, endothelial cells, and fibroblasts (Lin C.H. et al., 2013). Previous studies found that PARs could induce the cellular actions of thrombin and combine with thrombin to activate p38-MAPK/NF-**κ**B signaling (Lin H. et al., 2013). Peng *et al.* reported that hirudin could increase the viability of rat random skin flap and reduce the inflammatory response compared with the control group. The levels of p-p38/p38, p-NF-**κ**B p65/NF-**κ**B p65, TNF-**κ**, IL-6, and intercellular adhesion molecule-1 (ICAM-1) were significantly decreased in the hirudin-treated group. Hirudin has been proved to bind to PAR-1 of thrombin in previous studies (Greinacher and Warkentin, 2008), so it was speculated that hirudin might attenuate inflammation by inhibiting the interaction of thrombin with PARs (Peng et al., 2015). In brief, hirudin might block the PARs/p38/NF-**κ**B signaling pathway to improve random skin flap viability and reduce inflammation.

### JAK2/STATs

The Janus kinase/signal transduction and activator of transcription (JAK/STAT) pathway is crucial in various biological responses such as immune function, cell proliferation, differentiation and apoptosis. It has been reported that tumor cell apoptosis mediated by the activation of PAR-1 is associated with tyrosine phosphorylation of JAK2 and STAT1, and translocation of STAT1 to the nucleus (Huang et al., 2000). Hirudin could attenuate the apoptosis of human microvascular endothelial cells (HMVECs) and suppress the expression of p-JAK2 via antagonizing thrombin, suggesting that the underlying mechanism was related to the blockage of the JAK2/STATs signaling pathway. However, AG490, a tyrosine kinase inhibitor, also reduced the effect of thrombin, and the cell apoptosis rate was higher than that of the hirudin treatment, all of which indicated the presence of other signaling pathways besides the JAK2/STATs (Zhu et al., 2019). These results provided a novel scheme in relieving flap necrosis during the process of plastic and reconstructive surgery using random pattern skin flaps, but further study should be carried to elucidate other signaling pathways.

### VEGF/Notch

VEGF-Notch signaling pathway is a classical proangiogenic pathway that enables human pluripotent stem cells to differentiate into HMVECs (Sahara et al., 2015). After activated by related ligand, Notch signaling pathway releases Notch intracellular segments, which enter the nucleus to activate downstream target genes. And VEGF is an upstream promoter of Notch signaling pathway, which cooperatively participates in the process of angiogenesis (Wen et al., 2017). Previous studies proved that both natural hirudin and recombinant hirudin could significantly increase the VEGF expression in random skin flaps, which therefore improved random skin flap survival in rats. And natural hirudin showed more pronounced effects than recombinant hirudin (Yingxin et al., 2013). These findings provided a thread for the in-depth study of the molecular mechanism of hirudin in the treatment of skin flap necrosis. Recent studies investigated whether hirudin could promote the proliferation and angiogenesis of HMVECs through VEGF-Notch signaling pathway. It has reported that hirudin of different concentrations showed inverse effects on angiogenesis, that is, low and medium concentrations (1, 4 ATU/ml) of hirudin could promote angiogenesis, while high concentration (7 ATU/ml) showed inhibition effect. The expression of Notch1 was higher in 1 and 4 ATU/mL groups and lower in 7 ATU/ml group compared with control group. In addition, the expression of VEGF was higher in 1, 4, and 7 ATU/ml groups than in control group. These phenomena indicated that hirudin at low and medium concentrations could activate VEGF-Notch signaling pathway to promote the proliferation and angiogenesis of HMVECs. However, with the increase of drug concentration, VEGF-Notch signaling pathway was inhibited to induce HMVECs proliferation inhibition, which implied that high concentrations of hirudin tend to show anti-inflammatory property (Lin et al., 2018).

### VEGF/p38 MAPK/ERK1/2

P38 MAPK and ERK are the main family members of MAPKs, which play a crucial role in balancing angiogenesis inhibitors and angiogenesis promoters by regulating the expression of different transcription factors, such as AP-1 and c-Jun (Cowley et al., 1994). The AP-1 or c-Jun/AP-1 dimer has a recognition function, which can exert its own function by binding to the binding site of VEGF promoter sequence AP-1 on cell DNA coding. Increase of the AP-1 binding activity by MAPKs activation might lead to the activity of VEGF gene transcription. These studies provide a possibility that hirudin might regulate VEGF expression through its modulation of ERK and p38 MAPK activity (Xu et al., 2002). Hirudin has been shown to promote the neovascularization of ischemic skin flap in rats by inducing the expression of VEGF and down-regulating the expression of thrombin-induced thrombospondin-1 (TSP-1). And the underlying mechanism might be related to p38 MAPK and ERK1/2 signaling pathway (Gao et al., 2020). Another research proved these results, which demonstrated that the MEK inhibitor and the p38 MAPK inhibitor could attenuate the hirudin-induced VEGF expression and the TSP-1 expression, respectively. And SB203580, a specific inhibitor of p38 MAPK, could activate ERK1/2 in ischemic flaps, which indicated that cross-talk between p38 MAPK and ERK might exist in rat ischemic flap tissue. In addition, PAR1 antagonist, as well as hirudin, attenuated the p38 MAPK phosphorylation and increased the ERK1/2 phosphorylation, suggesting that the cross-talk modulated by thrombin/PAR1 signaling may participate in the process of hirudin-stimulated ERK1/2 signaling (Pan et al., 2015). In conclusion, these observations suggest that hirudin exerts the angiogenesis effect by regulating the expression of angiogenic and antiangiogenic factors via a cross-talk of p38 MAPK-ERK pathway.

### MCP-1/NF-κB

Monocyte chemoattractant protein-1 (MCP-1) is a major chemokine that chemotaxis attracts neutrophils and macrophages (Medina et al., 2005), which is secreted by fibroblast monocytes and vascular endothelial cells during the inflammatory process. High expression of MCP-1 can specifically chemotaxis monocytes/macrophages and promote the inflammatory process (Wetzler et al., 2000). Previous studies have revealed that the MCP-1 promoter contains two NF-κB binding sites (Peng et al., 2015), so altering the activation of the NF-κB pathway may modulate the secretion of MCP-1, thereby affecting the inflammatory response of tissues. Hirudin was shown to possess the effect of promoting wound healing in SD rats after laser surgery. Studies found that the wound area in the hirudin group was significantly smaller than that in the control group on the fourth and sixth day after operation. Moreover, the expression of MCP-1 wound area in the hirudin group was smaller than that in the control group, while the vascular density value (MVD) in the hirudin group was larger than that in the control group on the second and fourth day after operation. On the sixth day, the expression of MCP-1 and MVD in the hirudin group were similar to that in the control group (Qin et al., 2020). These results indicate that hirudin can reduce the release of MCP-1, promote tissue micro-angiogenesis, and promote wound healing in the early stage of laser trauma. But further research is needed to clarify whether the activation of NF-kB signaling pathway can directly activate MCP-1, or whether there are other upstream signals in the therapeutic course of hirudin on laser trauma.

### Oxygen Free Radicals

Oxygen free radicals participate in various physiological and pathological processes, which can exert completely inverse effects at different doses. Previous studies demonstrated that free radicals can cause damage to the skin flap in the process of ischemia reperfusion by attacking the biofilm and causing the peroxidation of polyunsaturated fatty acid lipid (Kirschner et al., 1997); topical application of natural hirudin could protect flap by scavenging free radical. Yin et al. reported that natural hirudin significantly reduced the damage and the local inflammatory response while increased tissue antioxidant capacity. Besides, hirudin also relieved the congestion of flap by reducing capillary permeability, preventing platelet and leukocyte adhesion, aggregation, and improving blood circulation. These results illustrated that the topical application of natural hirudin increased the concentration of target sites of local flap and would be an alternative drug treatment method (Guo-Qian et al., 2012). In addition, both natural and recombinant hirudin treatments led to the enhancement of superoxide dismutase (SOD) activity and the decrease of malonaldehyde (MDA) levels, which may potentially contribute to the hirudin-mediated effects of improving skin flap survival. However, natural hirudin is more potent in preventing flap necrosis than recombinant hirudin, probably due to the chemical difference and the purity of the reagents (Yingxin et al., 2013). Remarkably, these studies suggested that the wound repair activity of hirudin is associated with its ability to inhibit oxygen free radical damage.

## Anti-fibrosis Effect

Fibrosis refers to the pathological process of necrosis of parenchymal cells in organs, abnormal increase of extracellular matrix (ECM) in tissues and excessive deposition due to acute and chronic inflammation, which occurs in a variety of organs, such as liver, kidney, lung and heart (Xin et al., 2018). Unlike many different features of organs and tissues, fibrotic changes have a common pathogenic pathway throughout the body, which can be mainly summarized as initiation response driven by primary injury to the organ, the activation of effector cells, and the elaboration of extracellular matrix (Wynn, 2007; Urban et al., 2015). Nevertheless, despite the pathogenesis of fibrosis have been revealed to a certain degree, there are currently no approved treatments that directly target its mechanisms. Previous studies proved that the specific mechanisms of fibrosis regression include resolution of chronic tissue injury, transition from inflammatory process to recovery, inactivation of myofibroblasts, and ultimately fibrolysis of excess matrix scaffolds (Weiskirchen et al., 2019). Recently, multiple studies have reported that hirudin exerts remarkable anti-fibrotic effects on renal interstitial fibrosis (RIF), myocardial fibroblasts fibrosis, idiopathic pulmonary fibrosis (IPF), and other fibrosis diseases. Notably, the underlying molecular mechanisms involve the regulation of inflammatory and apoptotic cytokines, the reduction of ECM deposition, as well as the modulation of multiple signaling pathways such as TGF-β_1_/Smad/NF- κB, JAK/STAT3, ERK1/2, and PI3K/AKT. The molecular pathways connected with this pathology are as follows.

### TGF-β_1_/Smad/NF- κB

Transforming growth factor β_1_ (TGF-β_1_ /Smads signaling plays a significate role in activating myofibroblasts that overproduce ECM in tubular interstitial (Xing et al., 2017). In the canonical TGF-β_1_ signaling pathway, TGF-β_1_ can bind to its type II receptor (T RII)- kinase to activate its type I receptor (T RI)-kinase, which eventually lead to the activation and phosphorylation of downstream substrates of Smad2 and Smad3. Phosphorylated Smad2/3 can bind to Smad4 and translocate into the nucleus to regulate the target gene transcription (Song et al., 2017). Therefore, blocking TGF-β_1_ signaling pathway may be an effective strategy for fibrosis, such as RIF. Recently, Yang et al. confirmed that hirudin (10, 20, 40 IU/kg) could alleviate the renal damage and the deposition of ECM in renal interstitium of unilateral ureteral obstruction (UUO) rats (Yang et al., 2020). The expressions of TGF-β_1_, phosphorylated-Smad2, and phosphorylated-Smad3 in the kidneys of UUO rats were decreased, which illustrated the inhibition of TGF-β_1_/Smad signaling. Furthermore, the levels of several inflammatory markers (IL-6 and TNF-α), as well as the expression of P65, phosphorylated-P65, and phosphorylated-iκBα were down-regulated, which implied that the NF-κB signaling was implicated in the treatment of fibrosis. These findings were consistent with previous notion that NF-κB signaling is the classical pathway regulating inflammation, and the blockade of NF-κB signaling would improve renal fibrosis (Chung et al., 2019). Furthermore, the level of PAR-1 was decreased accompanied by downregulated signaling of TGF-β_1_ and NF-κB, which indicated that PAR-1 might act as the upstream trigger of these signaling pathways, but its specific role is required in future research. *In vitro*, TGF-β1 is an independent factor, which induces epithelial-mesenchymal transition (EMT) of renal tubular epithelial cells to become fibroblasts. Recently, Xie *et al.* found that hirudin (10–15 mg/kg) could decrease fibrosis, EMT, inflammation and cell apoptosis in renal tissues of UUO rats and TGF-β-induced renal tubular epithelial cells. It was shown that hirudin reduced the expression of inflammatory cytokines such as IL-1β, IL-6, and TNF-α, down-regulated the expression of related proteins of fibrosis and ECM including COL-I, FN, α-SMA, N-cad, and slug, while increased the expression of E-cad in mice serum and TGF-β-induced renal tubular epithelial cells. In addition, the expression of the apoptosis related proteins like pro-caspase3, pro-caspase9, Bcl-2 and Bax was also down-regulated in renal tissues of UUO rats after the administration of hirudin (Xie et al., 2020).

### JAK/STAT3

The JAK/STAT signaling pathway is involved in the pathogenesis of inflammatory and autoimmune diseases (Banerjee et al., 2017), whose activation is critical for glomerulosclerosis and renal tubulointerstitial fibrosis in diabetic nephropathy. Previous studies proved that inflammation mediated the initiation and underlying pathological process of renal interstitial fibrosis, thus inhibition of the JAK/STAT pathway could effectively reduce macrophage infiltration and reverse the inflammatory response to renal fibrosis (Bao et al., 2015). Fan *et al.* reported that hirudin (3, 10 mg/ml) could reverse the abnormal proliferation and fibrosis of HK-2 cell induced by TGF-β_1_ (5 ng/ml). In addition, the mRNA and protein expressions of α-smooth muscle actin (α-SMA), collagen-I (COL-I), fibronectin (FN), JAK and STAT3 were significantly down-regulated, which implied that the therapeutical effect of hirudin might be concerned with the inhibition of JAK/STAT3 signaling pathway, but the specific mechanism of its action still needs to be confirmed by further study (Fan et al., 2018).

### ERK1/2

As the main pathway of the MAPK system, extracellular signal-regulated kinase1/2 (ERK1/2) signal transduction pathway can transduce extracellular stimulation signals into the nucleus to cause various biological responses including oxidative stress, cell proliferation, apoptosis, and differentiation (Yoshioka, 2004). The ERK1/2 pathway has been reported in the development of cardiovascular disease including that of myocardial fibrosis, and activated ERK1/2 will initiate the activation of downstream signaling (Liu et al., 2012). A study in 2018 showed that hirudin could depress the myocardial fibroblasts induced by angiotensin II (Ang II) via inhibiting oxidative stress, regulating fibrosis-related factors, and repressing the ERK1/2 pathway in a dose-dependent manner. Remarkably, compared with the Ang-II group, phosphorylation level of ERK1/2 was decreased after hirudin administration. Besides, the reactive oxygen species (ROS) production, lactate dehydrogenase (LDH) activity, and MDA content were reduced by hirudin, while the SOD activity was enhanced. Moreover, the levels of several fibrosis-related factors such as matrix metalloproteinase-2 (MMP-2), MMP-9, FN, TGF-β_1_, COL-I, and COL-III were significantly down-regulated, but the expression level of tissue inhibitor of metalloproteinases-2 (TIMP-2) was upregulated (Yu et al., 2018).

### PI3K/AKT

IPF characterized by the formation of fibroblast focal, massive deposition of extracellular matrix, collagen accumulation and alveolar destruction, is a prototype of chronic, progressive, and fibrotic lung disease (Richeldi et al., 2017). PI3K/AKT pathway is considered to be one of the most important pathways in the course of IPF, which can be activated by upstream TGF-β_1_/Smad signaling (Hardie et al., 2010). Generally, AKT is the core of the PI3K/AKT signaling pathway and involved in the process of cell proliferation, differentiation and apoptosis after phosphorylation. Zhang *et al.* discovered that the expression of AKT and p-AKT protein were significantly increased in lung tissue of IPF rats induced by bleomycin, while hirudin could obviously alleviate these abnormal expressions and slow down the progression of IPF, which illustrated that hirudin was likely to act on IPF through the PI3K/AKT pathway (Zhang et al., 2019). However, these studies did not demonstrate the changes of upstream and downstream signaling molecules, and more studies are need, with focus on the key proteins in the cos-talk between TGF-β_1_/Smad and PI3K/Akt to clarify the effect of hirudin on IPF.

### PAI-1

Plasminogen activator inhibitor-1 (PAI-1) is an important component of the fibrinolytic system as well as a regulator of the plasminogen activator inhibitor system (PAs). The urokinase/tissue type plasminogen activator (uPA/tPA) and plasmin are indispensable in the cellular proteolytic degradation of ECM proteins and the maintenance of tissue homeostasis. Previous studies found that the activities of uPA/tPA/plasmin and plasmin-dependent MMPs mainly depend on the activity of PAI-1, a potent inhibitor of uPA/tPA (Ghosh and Vaughan, 2012). However, under pathologic conditions, excessive PAI-1 will counter the effects of uPA, and then contributes to excessive accumulation of ECM, which eventually promotes the occurrence and development of fibrosis (Kaji, 2016). Hirudin (25, 50, 100 ATU/ml) could exert therapeutic effect on PIF rats induced by bleomycin via inhibiting the expression of PAI-1 gene and protein. It was also found that the therapeutic effect of hirudin was significantly dose-dependent, and that hirudin combined with hormones was more effective in treating IPF in rats (Li et al., 2019). In short, Hirudin could reduce fibrin deposition and accelerate collagen degradation by reducing thrombin expression in the lung and by reducing PAI-1 content and activity. These findings provide a theoretical basis for clinical application of hirudin in the treatment of IPF, so as to broaden the treatment approach of IPF.

In recent years, the anti-fibrosis activity has also been observed in several derivatives of hirudin, and these derivatives showed better therapeutic effects than hirudin. Boronophenylalanine-modified hirudin is a new derivative of hirudin, which modified the site 63 of rH with the boronic-amino acid of boronophenylalanine through the codon-expanding method. Xin *et al.* reported that the antithrombin activity of rH was significantly increased after modified with boronophenylalanine. In addition, the proliferation inhibiting ability on fibroblast L929 cells was also obviously increased, which indicated that boronophenylalanine-modified hirudin might be used as a promising agent to treat fibrosis (Xin et al., 2015). Furthermore, rhSOD2-hirudin, a dual-feature fusion protein, fused by Hirudin and human manganese superoxide dismutase (hSOD2) could inhibit the proliferation of fibroblasts and reduce the Hydroxyproline (HYP) production *in vitro* by inhibiting the activity of thrombin. Besides, it could significantly decrease the lung inflammation and fibrosis in mice model of pulmonary fibrosis induced by bleomycin. These results indicated that increasing SOD2 levels to prevent fibrosis is an important effect of rhSOD2- Hirudin protein (Shen et al., 2019), and this protein possess the potential to be a candidate drug for pulmonary fibrosis.

## Effect on Diabetic Complications

Diabetes mellitus (DM) is considered the metabolic and inflammatory diseases associated with a range of microvascular, macrovascular and metabolic complications that were expected to affect 693 million adults by 2045 (Ahlqvist et al., 2015; Cole and Florez, 2020). The incidence and mortality of DM are on the rise worldwide mainly due to macrovascular complications and microvascular complications such as diabetic kidney disease, diabetic retinopathy as well as neuropathy. In the early stages of DM, intracellular hyperglycemia tends to bring about abnormal blood flow and increased vascular permeability, which implies reduced activity of vasodilators, increased activity of vasoconstrictors, and modulation of permeability factors such as VEGF. Subsequently, because of programmed cell death and further capillary occlusion induced by the overproduction of extracellular matrix as well as the deposition of extravasated periodic acid Schiff-positive plasma proteins, the loss of microvascular cell occurs, which eventually lead to various diabetic complications (Brownlee, 2001). Previous studies found that oxidative stress, inflammatory response, and cellular death play a pivotal role in the development of diabetes complications (Giacco and Brownlee, 2010; Volpe et al., 2018). Therefore, alleviating or blocking the process of these biological responses helps to prevent diabetic complications to a great extent. Hirudin has been reported to be useful to preventing diabetic nephropathy (DN) and diabetic peripheral neuropathy, and the potential molecular mechanisms mainly include the inhibition of HIF-1α/VEGF and p38 MAPK/NF-κB pathways, or the activation of NRF-2/HO-1 pathways.

### HIF-1α/VEGF

VEGF is a highly specific mitogen for endothelial cells, which induces endothelial cell survival, proliferation, and migration. While hypoxia-inducible factor 1 (HIF-1) is a heterodimeric transcription factor that mediates cellular responses to ischemic or hypoxic conditions via the transcriptional activation of specific genes such as VEGF (Wang et al., 2009). Numerous studies showed that the HIF-1α/VEGF signaling pathway was involved in the regulation of the ECM, and the increased deposition of ECM was of great significance in the pathogenesis of diabetic kidney disease (DKD) (Ando et al., 2019). Hirudin was reported to significantly improve renal function in the rat model of DKD, while suppress the expression of FN, type IV collagen, HIF-1α, and VEGF proteins. *In vitro*, hirudin could reduce the expression of ECM associated proteins in HK-2 human renal tubule epithelial cells induced by high glucose (HG), which was consistent with the results in previous studies (Zhou et al., 2012). These findings proved that hirudin reduced the expression of ECM markers by inhibiting the HIF-1α/VEGF signaling pathway in DKD renal tubular epithelial cells (Pang et al., 2020a). Interestingly, recent studies reported that hirudin/liposome complex showed a significant therapeutic effect on DN rat models by inhibiting the expression of VEGF, and this complex increased the accumulation of hirudin in kidney tissues compared with injections of hirudin alone (Wang et al., 2019). Probably, modification or preparation of hirudin to deliver drugs to specific organs may be helpful for further study of the pharmacological mechanisms.

### p38 MAPK/NF-κB

The MAPK signaling pathways modulate a diversity of cellular activities including survival, differentiation, proliferation and neuronal death (Kim and Choi, 2010). Whereas hyper-phosphorylation of MAPK molecules ultimately activates the transcription factor NF-κB and the subsequent production of inflammatory molecules (Saccani et al., 2002), which indicates that the NF-κB and p38 MAPK pathways are strongly associated with inflammation regulation. Previous studies discovered that inhibiting inflammation can protect the kidneys during DN, but it is not clear whether hirudin protects DN kidneys through NF-κB and p38 MAPK pathways (Wada and Makino, 2013). Recently, Han *et al.* found that hirudin could reduce macrophage infiltration, the expressions of proinflammatory cytokines (TNF-α, IL-1β, and IL-6), the expression of p-p38/p38 and p-p65/p65 protein, as well as the apoptosis of podocyte in the kidney tissues of DN rats induced by streptozotocin (STZ). Besides, the same phenomena were observed in HG-induced podocytes *in vitro*. The underling mechanism was connected with the inhibition of p38 MAPK/NF-κB signaling pathways in renal tissues and podocytes (Han J.et al., 2020). Another research pointed out that hirudin could reduce nephropathy microangiopathy in DN rats induced by STZ, and this might be concerned with the inhibition of glomerular endothelial cell migration and angiogenesis by down-regulating VEGF expression via blocking p38MAPK/NF-kB signaling pathway. Likewise, hirudin was shown to possess the capacity to decrease HG-induced activation of RhoA, as well as the expression of p-MYPT1/MYPT1and p-p38/p38 protein in glomerular endothelial cells (Pang et al., 2020b). Taken together, p38 MAPK/NF-κB signaling pathway may serve as a hub for inflammation and DN, which crosslinks with pivotal cytokines as well as kinase such as VEGF and RhoA to exert protective property in diabetic complications.

### Nrf-2/HO-1

As a redox-sensitive transcription factor, nuclear factor erythroid 2-related factor-2 (Nrf-2) plays a significant role in the primary defense mechanisms of oxidative stress and exogenous toxic substances (Kang, 2020). And among diverse antioxidant proteins regulated by Nrf-2, heme oxygence-1 (HO-1) is one of the most vital antioxidant stress detoxification enzymes, which exert positive effect in oxidant stress, inflammation, apoptosis and microcirculation (Hahl et al., 2013). Therefore, activation of Nrf-2/HO-1 pathway is of great importance for alleviating oxidant stress and inflammation. Recently, a research reported that hirudin could promoted the activity of dorsal root ganglion neurons (DRGn) under HG conditions by reducing the levels of ROS, up-regulating the expressions of the mRNA and protein of Nrf-2 as well as HO-1, which demonstrated that Nrf-2/HO-1 pathway was activated after hirudin administration. In addition, the expressions of NF-κB and Caspase-3 were suppressed in HG-treated DRGn by hirudin, indicating that NF-κB pathway and apoptosis also contributed to the therapeutic process (Liu et al., 2020).

## Anti-tumor Effect

Recently, multiple studies have confirmed the anti-tumor activity mediated by hirudin and revealed its mechanism to some extent. Hirudin has a wide anti-tumor spectrum and can be used against a variety of tumors, which include glioma, hepatocellular carcinoma, hemangioma, nasopharyngeal carcinoma (Liu et al., 2016) Tao et al., 2018), and others. Hirudin exerts its anti-tumor effect by inhibiting the proliferation, migration, and invasion of tumor cells, promoting the apoptosis, and modulating the cell cycle. The underlying mechanisms include down-regulating ERK/MAPK, VEGF/VEGF-R, and HGF/C-met signaling pathways, reducing the expression of angiogenesis related factors focal adhesion kinase (FAK), and others.

### ERK/MAPK

Glioma is one of the most common brain tumors, which accounts for 81% of intracranial malignant tumors (Ostrom et al., 2014). Although several treatments have been applied, the prognosis of patients with glioma is still poor. Previous studies proved that up-regulation of the ERK/MAPK pathway could amplify the mitogenic stimuli and promote the cellular proliferation of malignant gliomas (Han et al., 2016). Therefore, blocking this pathway may be a promising way to treat gliomas. Li *et al.* reported that hirudin exhibited antitumor effect when targeting cells of the LN229 and U251 cell lines *in vitro*. It significantly suppressed the growth of LN229 and U251 cell lines, with the *IC*_*50*_ of 30 and 15 mM, respectively. In addition, hirudin also increased the apoptosis in these glioma cells by down-regulating the expression of Bcl-2 and increased cell cycle arrest at the G1 phase. Notably, the content of pERK1/2 was decreased after hirudin administration, as well as the level of the protein Cyclin D1, which suggested that down-regulation of ERK/MAPK signaling pathway might be a crucial mechanism of hirudin in treating gliomas (Zhao, 2015).

### VEGF/VEGF-R

The abnormal angiogenesis is associated with numerous diseases such as inflammation, rheumatoid arthritis, and cancer (Kiselyov et al., 2007). At present, targeting angiogenesis is one of the most promising strategies for anticancer drug development. VEGF and VEGF receptors are pivotal proteins in modulating angiogenesis, and inhibiting the VEGF/VEGF-R pathway was proved to be efficient in cancer therapy (Sitohy et al., 2012). Recently, hirudin was found to possess inhibitory property on hepatocellular carcinoma, and the underlying mechanism might be implicated in suppressing angiogenesis. Li *et al.* reported that hirudin could inhibit the proliferation, apoptosis, migration and invasion of HepG2 cells in a dose-dependent manner. In addition, the expressions of VEGF mRNA and protein were significantly down-regulated after the treatment of hirudin (Li et al., 2016), which is consistent with previous studies that the reduction of hepatocellular carcinoma angiogenesis owes to the inactivation of VEGF pathway (Morse et al., 2019). Furthermore, hirudin is a specific inhibitor of thrombin, which can inhibit angiogenesis stimulated by thrombin and reduce the expression of VEGF. Hemangioma is a lesion caused by the abnormal proliferation of vascular endothelial cells and pericyte, which could be intervened and treated by antiangiogenic drugs (Ji et al., 2014). A study in 2015 revealed that 4 U/ml hirudin could effectively inhibit the proliferation and promote the apoptosis of mouse EOMA hemangioma cells *in vitro*, which showed an obvious dose-effect relationship (Yang et al., 2015).

Besides natural hirudin, rH was proved to have antitumor effects in Hep-2 human laryngeal cancer (LC) cells via suppressing angiogenesis. It was reported that the inhibitory effect of rH at 2 mg/ml was similar to that of DOX (a standard widely used clinical chemotherapy drug) at a concentration of 4 μg/ml. rH inhibited the cell viability and induced apoptosis in LC Hep-2 cells by reducing the level of Bcl-2, while increasing the content of pro-apoptotic protein Bad. Remarkably, rH significantly down-regulated the expression of proteins FAK and VEGF-R, suggesting that the anti-tumor effect of rH might be associated with the suppression of angiogenesis by down-regulating the VEGF/VEGF-R pathway. (Lu et al., 2015). These findings also suggested that targeting thrombin by rH is a potential strategy for treating LC. In addition, rH also exerts anti-tumor effect when targeting human hepatocellular carcinoma SMMC-7721 cells, which not only suppressed the proliferation and enhanced the apoptosis of SMMC-7721 cells, but also reversed the carcinogenesis effects by thrombin (Wu and Li, 2015).

### HGF/C-Met

Hepatocyte growth factor (HGF) as well as its receptor, mesenchymal epithelial transition factor (c-Met), are involved in the occurrence and development of various tumors. Once c-Met is activated, the cell proliferation, invasion, survival, angiogenesis, and motility will be enhanced, therefore, abnormal increase of c-Met could be observed in several solid tumors (Miranda et al., 2018). Intervening the expression of HGF/c-Met signaling was confirmed to be a valid method to inhibit the malignant transformation, angiogenesis, growth and invasion of tumors (Wang et al., 2020). Recent studies manifested that hirudin has a certain therapeutic effect on hepatocellular carcinoma (HCC). Yao *et al.* reported that hirudin could exert significant inhibition effect on H_22_ tumor cells in mice, with the inhibition rates of the high-dose hirudin group (2.0 g/kg) and the low-dose hirudin group (1.0g/kg) were 37.63 and 23.71%, respectively. Additionally, the expression of C-met was obviously decreased in hirudin treatment groups, which implied that the underlying mechanism might be related to the suppression of the HGF/c-Met signaling pathway (Yao et al., 2011). Besides, hirudin could also exert inhibitory property in laryngeal carcinoma tumor-bearing mice through affecting that pathway. The inhibition rates of 1.2g/kg and 2.5 g/kg hirudin-treated groups were 40.1 and 26.92% respectively. Meanwhile, the expression of c-Met in tumor tissues was obviously down-regulated after hirudin administration (Ma et al., 2017). These researches suggested that inhibiting the HGF/c-Met signaling pathway might be an important mechanism of hirudin in hepatocellular and laryngeal carcinoma therapy.

## Anti-Hyperuricemia Effect

Hyperuricemia (HUA) is a metabolic disorder of purine metabolism, which can lead to the increase of uric acid in blood. Individuals with hyperuricemia might develop gout, a painful inflammatory arthritis caused by monosodium urate crystal deposition in articular structures (Lu et al., 2019). At present, the pathologic mechanisms of hyperuricemia have not been fully elucidated, and related studies mainly focus on the synthesis and the excretion of uric acid. It was proved that the main target affecting uric acid synthesis was xanthine oxidase, the key enzyme in uric acid synthesis (Xu et al., 2015). In addition, the uric acid transfer factor such as glucose transporter 9 (GLUT9), novel urate transporter 1 (URAT1), and organic anion transporters (OAT) in renal tubules are the crucial factors affecting uric acid excretion (Preitner et al., 2009). Hirudin could significantly reduce the level of uric acid in serum and urine of hyperuricemia rats induced by potassium oxonate. Besides, the expressions of GLUT9, URAT1 mRNA and protein were significantly reduced, while the expressions of OAT1 mRNA and protein were significantly increased. These results illustrated that hirudin could reduce the uric acid by regulating the expressions of renal urate transporters OAT1, URAT1 and GLUT9 in hyperuricemia rats (Wu et al., 2020). Liu *et al.* found that hirudin could significantly reduce the serum uric acid level in hyperuricemia mice induced by hypoxanthine and inhibit acute toe swelling induced by sodium uric acid in rats. In addition, the level of serum hyperuricemia and blood urea nitrogen (BUN), as well as the expression of GLUT9 were decreased in chronic hyperuricemia mice induced by potassium oxonate after hirudin administration, which manifested that hirudin could exert obvious anti-hyperuricemia and anti-gout effects by regulating the expression of GLUT9 and protecting kidney (Liu et al., 2018).

## Effect on Cerebral Hemorrhage

Intracerebral hemorrhage (ICH) is a cerebrovascular disease with high mortality and morbidity, but the effective treatment is still lacking. Previous studies indicated that ICH-induced brain injury is not only due to the effect of hematoma mass and the expansion of potential hematoma but also due to secondary brain injury (SBI) (Chen et al., 2015). After SBI, the permeability of blood-brain barrier is significantly increased, which can lead to the leakage of a large number of contents in brain cells and the rapid increase of water content in brain, eventually resulting in the formation of brain edema (Kim and Bae, 2017). Besides, apoptosis of brain cells is one of the mechanisms of SBI (Wang et al., 2018). Local application of hirudin in rats after cerebral hemorrhage could reduce the tissue edema and inhibit the apoptosis of nerve cells, which mainly characterized by the alleviation of cell necrosis and tissue edema around the hematoma, the up-regulation of Bcl-2, and the down-regulation of Caspase-3 in brain (Yin and Wei, 2013). Another research demonstrated that hirudin could reversed the abnormal increase of brain water content, brain coefficients, and brain cell apoptosis rate in cerebral hemorrhage rats induced by injecting autologous arterial blood into the caudate nucleus. In addition, the expression levels of p-JAK2 and p-STAT3 in hirudin group were lower than those in the model group. These results illustrated that hirudin could reduce apoptosis of brain cells after intracerebral hemorrhage in rats and the protective mechanism may be associated with the inhibition of JAK2/STAT3 signaling pathway (Li et al., 2017).

## Other Effects

IgA nephropathy (IgAN) is a leading cause of CKD and renal failure, which is characterized by active lesions, active lesions and interstitial fibrosis (Rodrigues et al., 2017). Previous studies reported that the activation of pro-inflammatory and pro-fibrotic mediators is one of the most significant features of IgAN, and blocking the inflammatory reaction and kidney interstitial fibrosis may be a crucial target in the treatment of IgAN (Zhang et al., 2015). Recent results revealed that hirudin could ameliorate IgAN through suppressing fibrosis and inflammatory response. It was shown that hirudin could reverse the increased level of proteinuria, serum creatinine and urea nitrogen in IgAN rats. In addition, the elevated number of apoptotic bodies and expressions of caspase-3 as well as caspase-9 were alleviated by hirudin. Besides that, the fibrosis indexes such as TGF-β_1_, CoI-IV, and fibronectin-1 were remarkably suppressed in IgAN rats after hirudin administration. Moreover, the levels of inflammatory factors (IL-1b, IL-6, and IL-18) were reduced in hirudin-treated group. Notably, several pathways including NF-κB, TNF-α, IκBα, and VCAM-1 were inhibited in the therapeutic process of hirudin (Deng et al., 2019). These findings might provide a new therapeutic method to treat IgAN. Furthermore, hirudin could exert protective effect in rat with acute lung injury. The abnormal expression of PAR-1 mRNA of lung tissue in acute lung injury rat induced by endotoxin was significantly declined after the treatment of hirudin, as well as the content of TNF-α and MMP12. These phenomena indicated that hirudin could inhibit the inflammation and fibrosis in acute lung injury rat and play a protective role as an anti-inflammatory mediator (Bao et al., 2012). Moreover, hirudin was shown to possess therapeutic property in isoproternol-induced myocardial infraction. The levels of myocardial Creatine Kinase Isoenzyme-MB (CK-MB), LDH, ROS, and MDA were apparently decreased in myocardial infraction (MI) rats after hirudin administration. Besides, the expressions of Nrf2-dependent gene (HO-1, SOD) were effectively upregulated, which implied that hirudin could induce the activation of Nrf2 signaling pathway. Furthermore, hirudin could restore mitochondrial membrane potential in addition to cytochrome C-related apoptosis (Zhang et al., 2020).

### Pharmacokinetics

Pharmacokinetic studies of hirudin and rH have been conducted in many species, including rats, rabbits, dogs, and human; the detailed pharmacokinetic parameters of these studies are shown in Table 2. Due to the polypeptide structure of hirudin, it is difficult to achieve the effective concentration by oral administration. Thus, hirudin is usually administered parenterally for high bioavailability. The pharmacokinetic behavior of hirudin among different animals tend to be the same. After intravenous administration, the kinetics of hirudin manifests as a two-compartment open model (Markwardt et al., 1988b; Richter et al., 1988; Kaiser et al., 1990). The absorption half-life (t_1/2α_) suggested that hirudin can be rapidly distributed from the central compartment to the peripheral compartment. In addition, the elimination half-life (t_1/2β_) was about 1 h, which indicated that hirudin can be quickly excreted and metabolized. Besides, the bioavailability of hirudin was almost 100% after subcutaneous administration, and the concentration-time curves illustrated that its plasma pharmacokinetics was consistent with a one-compartment model (Nowak et al., 1988). Compared with intravenous administration, the elimination half-life (t_1/2_) was obviously prolonged in both rats (2.1 h) and dogs (3.03 h). Similar to natural hirudin, rH could be rapidly distributed into the extravascular compartment with t_1/2α_ from 0.08 to 0.25 h. In addition, the elimination phase half-lives were relatively longer after intravenous administration. Remarkably, most of the administered hirudin and rH could be eliminated through kidney in active form in dogs (Nowak et al., 1988). Moreover, in healthy volunteers, renal clearance and degradation accounted for 90% of systemic clearance (Greinacher and Lubenow, 2001).

**TABLE 2:** Pharmacokinetic studies of natural hirudin and recombinant hirudin.

Route of administration	Species	Dose	Pharmacokinetic parameters											References
			t_1/2α_ (h)	t_1/2β_ (h)	k_12_ (h^−1^)	k_21_ (h^−1^)	k_10_ (h^−1^)	AUC_(0-t)_ (μg·h/mL)	V_c_ (L/kg)	V_dss_ (L/kg)	Cl_tot_ (ml/min)	T_max_ (h)	C_max_ (μg/ml)	
**Natural hirudin**														
i.v	Rats	1.0 mg/kg	0.21	1.08	0.59	0.65	1.48	1.74	0.116	0.222	2.833	-	-	Richter et al. (1988)
	Dogs	0.5 mg/kg	0.17	0.95	1.37	1.46	1.99	1.43	2.2	4.4	178	-	-	
	Rabbits	1.0 mg/kg	0.11	1.16	-	-	-	1.31	0.10	0.23	-	-	-	Kaiser et al. (1990)
	Human	1000 ATU/kg	-	0.84	-	-	-	5.69 ATU·h/mL	-	12.9	230	-	-	Markwardt et al. (1984)
i.h	Rats	1.0 mg/kg	-	2.10^a^	-	-	-	-	-	-	-	1.0	0.84	Markwardt et al. (1982)
	Dogs	0.5 mg/kg	-	3.03^a^	-	-	-	-	-	-	-	2.0	0.30	
**Recombinant hirudin**														
i.v	Rats	1.0 mg/kg	0.15	1.07	1.93	1.68	1.8	1.64	0.31	0.66	3.05	-	-	Markwardt et al. (1988a)
		2.0 mg/kg	0.08	1.18	4.15	1.55	3.14	3.81	0.17	0.63	2.09	-	-	
	Rabbits	1.0 mg/kg	0.11	1.16	2.67	1.21	3.24	4.31	0.07	0.23	12.38	-	-	Markwardt et al. (1989)
	Dogs	0.5 mg/kg	0.25	1.22	0.57	0.81	1.96	1.54	0.18	0.28	183	-	-	Nowak et al. (1988)
	Human	0.1 mg/kg	0.15	1.19	1.93	1.68	1.8	0.54	4.4	8.9	168	-	-	Nowak (1991)
nasal	Rats	6.0 mg/kg (spray)	-	1.20^a^	-	-	-	1.59	-	-	-	1.0	0.409	Zhang et al. (2006)
		4.0 mg/kg (Liposome)	-	1.39^a^	-	-	-	1.33	-	-	-	1.5	0.211	Zhang et al. (2007)
		30.0 mg/kg (Solution)	-	1.41^a^	-	-	-	1.48	-	-	-	2.0	0.375	
Intratracheal		10.0 mg/kg	-	4.71^b^	-	-	-	3.77	-	-	-	0.4	0.96	Liu et al. (2005)
Buccal		40.0 mg/kg	-	3.64^b^	-	-	-	0.63	-	-	-	2.5	0.15	
Rectal		40.0 mg/kg	-	1.98^b^	-	-	-	0.58	-	-	-	0.6	0.26	

^a^ The one-compartment model.

^b^ The non-compartmental model; Cl_tot_, total clearance from plasma; AUC, area under the plasma concentration time curve; V_c_, the apparent volume of distribution; V_dss_, the volume in steady state; C_max_, the peak plasma concentration; T_max_, the time after dosing when C_max_ occurred.

In addition to subcutaneous administration and intravenous administration, other routes of rH administration have been investigated. The *in vivo* course of rHV2 in rats fitted to the one-compartment model after intranasal administration of rHV2 spray with the relative bioavailability of 28.53% (Zhang et al., 2006). Furthermore, Zhang *et al.* reported that the relative bioavailability (FR) of rHV2 liposome and rHV2 saline solution after intranasal administration were 12.36 and 1.83%, respectively. These results demonstrated that the pharmacokinetics and bioavailability of rH were greatly affected by the dosage form (Zhang et al., 2007). Moreover, Liu *et al.* compared the pharmacokinetics of rH in rats administered through four different routes (intratracheal, buccal, nasal and rectal) and found that the pulmonary route was more suitable for systemic delivery of rHV2 than other three routes (Liu et al., 2005).

### The Preparations of Hirudin

In order to reduce the adverse reactions and improve the bioavailability of hirudin, various types of preparations have been reported in recent years, such as micelles, nanoparticles, TiO_2_ nanotube systems, polyamides dendrimer, and so on. Remarkably, the characteristics of these novel preparations mainly focus on prolonging circulation, thrombus targeting, continuous drug delivery, and specific disease targeting.

### Prolonging Circulation

Hirudin can be rapidly distributed into the intercellular space after intravenous injection and has a short half-life; repeated injections are needed to maintain its therapeutic effect. However, several drawbacks (such as high price and bleeding risk) will be brought about after repeated injections, which greatly limited the clinical application of hirudin. The bovine serum albumin (BSA) nanoparticles are considered to be a promising carrier to address those problems (Green et al., 2006). In 2015, hirudin-BSA nanoparticles have been successfully synthesized and characterized by a desolvation technique. These nanoparticles possess good encapsulation and certain sustained-release capacity, which could control the release of hirudin to prolong the antithrombotic effect, suggesting that hirudin-BSA nanoparticles might be applied in clinic therapy for thrombosis. However, the pharmacokinetics and safety of these novel preparations need to be further studied (Jing et al., 2016). In addition to nanoparticles, polydopamine fitted TiO_2_ nanotube systems were also exploited to extend the release of hirudin. In 2018, Yang *et al.* reported that PDA fitted TiO_2_ nanotube systems could prolong the release of bivalirudin and improve the hemocompatibility *in vitro* and *in vivo*. Besides, the systems could effectively reduce the formation of thrombosis by inhibiting the denaturation as well as adhesion of platelets, fibrinogen, and other blood components (Yang et al., 2018). Although hirudin could exert platelet aggregation inhibitory effect after structurally modified with platelet-targeting peptides such as RGD, the short half-life of this derivative is still a severe problem. Polyion complex (PIC) micelles have attracted extensive attention in recent years due to its complementary and unique features (Yang et al., 2009), which enables the long circulation of drugs *in vivo*. Recombinant hirudin variant 2 (rHV2)-loaded PIC micelles, consisting of methoxy poly (ethylene glycol)-grafted-chitosan (mPEG-g-chitosan) and Arg-Gly-Asp conjugated poly (ethylene glycol)-grafted-chitosan (RGD-PEG-g-chitosan), were prepared by Wang *et al.* in 2010. These rHV2-loaded PIC micelles, with the mean size of 41.9 ± 1.8 nm and the encapsulation efficiencies of 81.08 ± 0.85%, could prolong the mean retention time of rHV2 and specifically bind to platelets. Besides, efficient anticoagulant effects and platelet aggregation inhibition effects were observed in these micelles, which indicated that RGD-PIC micelles could achieve the purpose of platelet-targeted delivery and long circulation of rHV2 (Wang et al., 2010).

### Targeting Thrombus

Furthermore, a novel nonviral gene delivery system for thrombus targeting therapy was explored by Chen *et al.* in 2019. Based on PEGlyation polyamides dendrimer (PAMAM), this delivery system is modified with RGDyC to condense the pDNA with recombinant hirudine (rHV) gene (RGDyC-rHV-EGFP), which achieved the targeted enrichment and activation of the hirudine fusion protein in the thrombus site, improved the concentration of drugs in the thrombus site, as well as reduced the risk of bleeding (Chen J. et al., 2019). In 2020, Xu *et al.* exploited a closed-loop hirudin delivery platform (designated as HV/ctNGs) using self-regulated nanoscale polymeric gels for anticoagulant therapy, which significantly increased the stability and bioavailability, while reduced the clearance of hirudin *in vivo*. Besides, the accumulation of hirudin was also greatly increased in the fibrous clots due to the clot-targeted ligand. Remarkably, the engineered nanogel was able to adaptively release hirudin in response to changes in thrombin during the thrombosis pathological process, which observably increased the anticoagulant activity of hirudin and reduced its adverse effects (Xu et al., 2020).

### Targeting Specific Disease

In 2014, Sellers *et al.* prepared depot-formulations of Poly (lactic-co-glycolic) acid (PLGA) microsphere and Pluronic F-127 for continuous local delivery of hirudin. This controlled delivery could delay the release of hirudin, accelerate the recovery of motor function and increase the populations of oligodendrocyte in mouse treated post-injury, which might be applied to relieve scarring and promote regeneration in the central nervous system (Sellers et al., 2014). In addition to target nerve damage, the preparation of hirudin could play an important role in DN targeted therapy. In 2019, Wang *et al.* reported that injections of the hirudin/liposome complex could increase the accumulation of hirudin in kidney tissues in DN rat models compared with injections of hirudin alone, and this complex significantly alleviated renal injury in DN rat by down-regulating the expression of TGF-β1 and VEGF. These findings demonstrated that hirudin loaded by liposome could realize the targeted therapy of DN (Wang et al., 2019). Preparations of hirudin and its derivatives mentioned above are shown in Table 3.

**TABLE 3:** The preparations of hirudin and its derivatives.

Preparation	Effective constituent	Composition	Effects/applications	References
rHV2-loaded RGD-PIC micelles	rHV2	mPEG-gchitosan; RGD-PEG-g-chitosan	Platelet-targeted delivery; long circulation of rHV2	Wang et al. (2010)
Hirudin-BSA nanoparticles	Hirudin	BSA nanoparticles	Prolonged antithrombotic effect	Jing et al. (2016)
PDA fitted TiO_2_ nanotube systems	Bivalirudin	PDA; TiO2 nanotube arrays	Local drug delivery over an extended period	Yang et al. (2018)
RGDyC-mPEG-PAMAM	RGDyC-rHV-EGFP	PEGlyation polyamides dendrimer	Targeting therapy of thrombus	Chen Y. et al. (2019)
HV/ctNGs	rHV3	Self-regulated nanoscale polymeric gels	Increased anticoagulant activity; clot-targeted property	Xu et al. (2020)
Hirudin-loaded PLGA/F-127 gels	Hirudin	Poly (lactic-co-glycolic) acid microspheres; F-127 hydrogel	Continuous local delivery of hirudin; accelerating functional recovery from a demyelination lesion in the spinal cord	Sellers et al. (2014)
Hirudin/liposome complex	Hirudin	Liposome	Targeted therapy of DN	Wang et al. (2019)

#### Toxicity

The toxic studies of hirudin and its derivatives have been performed in several animal experiments. Previous studies revealed that after subcutaneous injection of hirudin 100 mg/kg for 8 days, the hemorrhage of pleura, pia meninges and peritoneum occurred in rats. In addition, daily doses of hirudin from 1 mg/kg to 5 mg/kg resulted in a dose-dependent increase in bleeding propensity in sub-chronic toxicity studies in rats and dogs for up to 3 months. It's worth noting that the formation of antibodies could be observed after the administration of extremely high doses of hirudin in dogs, but these antibodies did not neutralize the effect of hirudin (Nowak, 2002). Interestingly, such antibodies were also detected in humans during long-term administration of hirudin (Eichler et al., 2000), however, the antibody-bound hirudin could exert antithrombin property as before with the longer half-life, indicating that these antibodies might be a form of hirudin storage. Moreover, the lethal dose (*LD*_*50*_) of lyophilizing hirudin powder was determined to be over 10.0 g/kg when administered via orally administration, and no significant difference was observed in micronucleus rate and sperm malformation rate between hirudin groups (2.5, 5.0, and 10.0 g/kg) and control group (Huang et al., 2010). Furthermore, lyophilizing hirudin powder exhibited no maternal toxicity, embryo toxicity, and teratogenicity in rats at the dosages of 312.5, 1250, and 5000 mg/kg (Huang et al., 2011). Besides, the toxicity of rH has been investigated in some reports. Lu *et al.* discovered that rH (1.0, 3.0, 6.0 mg/kg) could significantly prolong the clotting time, thrombin time, and activated partial thromboplastin time in *Macaca mulatta*, however, these effects could be automatically reversed at 24 h after administration (Lu et al., 2004). These studies proved that hirudin rarely causes toxicity despite its minor bleeding side effect, which can be used for the prevention and treatment of more diseases with a broad development prospect.

## Conclusion and Discussion

In the past few decades, notable findings of hirudin have been gained, precisely including the clinical application, development of derivatives, exploration of novel pharmacological activities, and investigation of new preparations.

### Successful Application of Derivatives of Hirudin in Clinic

With the development of genetic engineering technology, the producing problems of natural hirudin has been successfully solved, which greatly promoted the successfully application of lepirudin, desirudin and bivalirudin in clinic. Lepirudin has been approved as an anticoagulant for patients with heparin-induced thrombocytopenia (HIT), with the usual dose of 0.4 mg/kg IV bolus followed by 0.15 mg/kg/h IV infusion (Greinacher and Lubenow, 2001). It could reduce HIT related adverse events by 72%, providing not only effective anticoagulant therapy, but also rapid platelet recovery in HIT patients. Noticeably, the bolus and infusion rates should be scheduled according to the serum creatinine values of patients (Greinacher et al., 1999). Desirudin is used to preventing deep venous thrombosis (DVT) in patients undergoing total hip replacement (THR) and total knee replacement (TKR) surgery by subcutaneously (SC) injection. Previous studies reported that desirudin was superior in preventing VTE in patients undergoing elective hip replacement surgery at the dose of 15 mg (Q12H, SC) compared with low-dose unfractionated heparin and low-molecular-weight heparin (Eriksson et al., 1997) (Bergese et al., 2013). Another direct thrombin inhibitor, bivalirudin, is effective on preventing thromboembolic complications. It was reported that administrating bivalirudin as a 1.0 mg/kg IV bolus, followed by 2.5 mg/kg/h by IV infusion for 4 h early in the trial; later, lowering the bolus to 0.75 mg/kg, followed by a 1.75 mg/kg/h infusion over 4 h achieved procedural and clinical success in 98 and 96% of the patients, respectively (Sun et al., 2017). Additionally, bivalirudin is also being studied in phase II and III multicenter trials as an alternative anticoagulant for both on-pump and off-pump cardiac surgery for patients with or without HIT (Joseph et al., 2014).

### Development of Derivatives

In recent years, studies on the anti-thrombotic effect of hirudin has transferred to its derivatives, mainly because the structural modification of hirudin brings in increase of the therapeutical activities and decrease of the adverse reactions. Compared with natural hirudin, the derivatives such as EPR-hirudin, GVYAR-hirudin, LGPR-hirudin, neorudin, and hAvHA could obviously decrease the bleeding risk by precisely targeting the thrombosis site. These studies indicated that recognition peptides identified from the blood coagulation factors (FXIa and FXa) are the key to enable hirudin targeting thrombus sites. FXIa, located upstream of the coagulation pathway, is a serine protease homodimer in the intrinsic pathway, which recognizes diverse peptide sequences such as EFSRVVG and EPR (Castillo et al., 1983; Scott et al., 1986). In addition, it can catalyze the activation of factor IX into factor IXa, which subsequently activates factor X to FXa (Al-Horani and Afosah, 2018). Located at the convergence point of the intrinsic and extrinsic pathways, FXa is a significant determinant of the procoagulant activity of whole blood clots and arterial thrombosis (Zhu et al., 2015), which exhibits its functional effect by recognizing sequences including GVYAR, IQGR and IEGR. Fusing these recognition peptides with the N-terminus of hirudin can lead to an impermanent loss of antithrombotic activity; nevertheless, once the coagulation system is activated, the antithrombotic activity will be recovered by cleavage of the corresponding factors (Zhang et al., 2008). In this way, hirudin can be delivered to thrombus site, ultimately avoiding the systemic bleeding complications. Additionally, other derivatives like recombinant-RGD-hirudin and DTIP can compensate for the reduced antithrombotic activity of recombinant hirudin. Previous studies illustrated that hirudin did not had any inhibitory effect on ADP-induced platelet aggregation (Mo et al., 2009). However, the recombinant-RGD-hirudin and DTIP can competitively inhibit the binding of fibrinogen to glycoprotein IIb/IIIa of platelets to inhibit the platelet aggregation. These results suggested that such bi-functional hirudin derivatives with improved efficacy and safety are worth of further research and would be a substitute for heparin in the treatment of thrombus in the future.

### Exploration of Novel Pharmacological Activities

**FIGURE 2:**
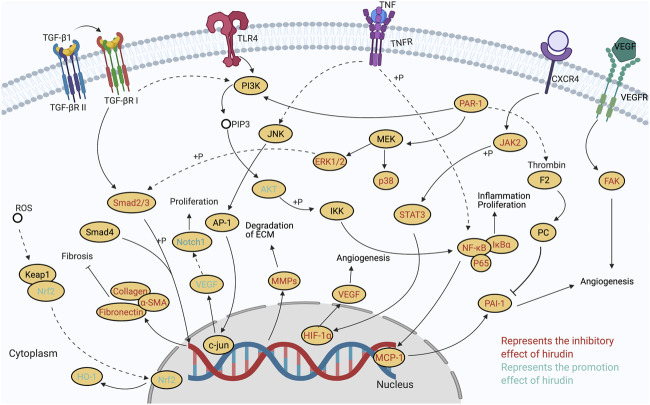
Molecular pathways involved in the pharmacological activities of hirudin.

In addition to the antithrombotic effect, some novel bioactivities of hirudin are explored in recent studies, particularly its roles in wound repair, anti-fibrosis, preventing diabetic complications, and anti-tumor. Besides, the effects on hyperuricemia, cerebral hemorrhage, IgA nephropathy, acute lung injury, as well as myocardial infraction have been also reported. Based on these pharmacological studies, it can be concluded that the therapeutic properties of hirudin are associated with its capacities of alleviating oxidative stress, suppressing inflammation, regulating apoptosis, promoting neovascularization, inhibiting thrombin, and others. Further studies on molecular mechanisms suggested that multiple signaling pathways are involved in these various therapeutic processes. The molecular and cellular targets of hirudin mentioned in this review are portrayed in Figure 2 **.** It can be inferred that the wound repair effect of hirudin is related to the inhibition of (PARs)/p38/NF-**κ**B, the suppression of JAK2/STATs, the activation of VEGF/Notch and ERK, and the repression of MCP-1. Regulation of these pathways results in increased expression of VEGF protein and increased phosphorylation of ERK1/2, decreased expression of pro-inflammatory cytokine, ICAM-1, TSP-1, MCP-1, and phosphorylation of p38, NF-**κ**B, and JAK2. In addition, hirudin-mediated treatment of diabetic complications is associated with the suppression of HIF-1α/VEGF and p38 MAPK/NF-**κ**B, and the activation of Nrf2/HO-1, which eventually lead to the downregulation of VEGF, HIF-1α, ROS, and caspase-3, while the increase of Nrf2 and HO-1. Furthermore, by inhibiting the signaling pathways including TGF-β_1_/Smad/ NF-**κ**B, JAK/STAT3, ERK1/2, PI3K/AKT, and PAI-1, hirudin plays a significant part in treating fibrosis. Accordingly, several key proteins such as TGF-β_1_, α-SAM, PAR-1, FN, COL, and PAI-1 involved in the process of fibrosis were down-regulated. The potential mechanisms of hirudin in preventing tumor include inhibiting the proliferation, migration, and invasion of tumor cells, promoting cell apoptosis, as well as modulating the cell cycle of tumor cells. Meanwhile, signaling pathways such as ERK/MAPK, HGF/c-Met, and VEGF/VEGFR also participated in the tumor therapy of hirudin. Besides, the potential mechanisms of other pharmacological effects associated with hirudin, such as preventing hyperuricemia, cerebral hemorrhage, IgA nephropathy, acute lung injury, and myocardial infraction, need to be further explored. These increasing researches indicate that hirudin may be a potential drug for the treatment of skin flap necrosis, fibrosis, diabetic complications, and cancers, which deserves further studies in the future.

### Investigation of New Preparations

Studies showed that novel preparations can further improve the bioavailability of hirudin and the derivatives. Preparations such as hirudin-BSA nanoparticles, PDA fitted TiO_2_ nanotube systems and rHV2-loaded PIC micelles could prolong the circulation of hirudin *in vivo*, which overcome the bleeding risk brought from repeated injections and makes up for the short half-life. In addition, hirudin delivery platforms like RGDyC-rHV-EGFP and HV/ctNGs realized thrombus targeting therapy by promoting the accumulation of hirudin in the fibrous clots. Furthermore, part of the preparations (Hirudin-loaded PLGA/F-127 gels and hirudin/liposome complex) can also target other diseases including nerve damage and DN, which provide potential for studying the molecular mechanism of hirudin in the treating different diseases.

#### Future Perspectives

Although the studies of hirudin have referred to pharmacy, pharmacology, preparation and clinical application, we find that there is still somewhat inadequate, especially studies of the dose-effect relationship, toxicity and safety of its derivatives and preparations. In this section, we focus on potential future directions for research on hirudin.

Given the multiple pharmacological activities nature of hirudin, elaborating its dose-effect relationship is beneficial to the determination of doses for clinical treatment of different diseases in the future. Remarkably, hirudin exhibited different activities and even opposite biological effects at different concentrations. For instance, the expression of VEGF was increased when hirudin was used to treat necrosis of skin flap, but VEGF was inhibited in hirudin–treated diabetic complications and tumor. This difference may be caused by the different doses of hirudin used in these two studies. At low concentrations (1 and 4 ATU/ml), hirudin can promote cell proliferation and micro-angiogenesis by upregulating VEGF, which is quite important for wound repair. However, with higher concentration (7 ATU/ml), the promoting effect of hirudin on the VEGF/Notch signaling pathway gradually decreased and turned into an inhibitory effect. Accordingly, the expression of VEGF decreased, indicating that high concentration of hirudin can inhibit the growth of cells and exert the anti-inflammatory activity, which is conducive to the treatment of diabetic complications and tumor. Likewise, the effect of hirudin on ERK1/2 pathway is also bidirectional. At the dosage of 2ATU, hirudin could significantly elevate the expression level of ERK1/2 phosphorylation; activated ERK promoted the transcription of VEGF signaling molecule by regulating the expression of AP-1 and c-Jun, which facilitated the neovascularization of ischemic skin flap rats. However, at other dosages (20, 40, and 80 μg/ml), hirudin could down-regulate the expression of ERK1/2 phosphorylation to participate in the treatment of fibrosis. Detailed information on the pharmacological activity, route of administration, effective dose and molecular mechanism of hirudin is provided in Table 4. It is worth noting that besides concentrations, different routes of administration and the animal models or cells used in different studies may contribute to the differences in bioactivities as well. Therefore, more *in vivo* researches are quite needed to clarify the dose-effect relationship of hirudin.

**TABLE 4:** Molecular mechanisms to the pharmacological activity of hirudin and rH.

Models/Cells	Administration route	Effective dose/concentrations	Molecular mechanisms	References
**Wound repair effect**				
Ischemic skin flap model in rats	Subcutaneous injection	6 ATU	Inhibiting the PARs/p38/NF-B signaling pathway	Peng et al. (2015)
		6 U	Increasing the mRNA and protein expression levels of VEGF.	Yingxin et al. (2013)
		2 ATU	Up-regulating the expression of VEGF and down-regulating the expression of TSP-1 via regulating p38 MAPK and ERK1/2 signaling pathways	Gao et al. (2020)
		2 ATU	Regulating the expression of angiogenic and antiangiogenic factors via p38 MAPK-ERK pathway	Pan et al. (2015)
Thrombin-induced HMVECs apoptosis	-	2 ATU/ml	Inhibiting the JAK2/STATs signaling pathway	Zhu et al. (2019)
Laser-induced skin wound injury in rats	Transdermal administration	20 ATU	Inhibiting the MCP-1/NF-κB signaling pathways	Qin et al. (2020)
HMVECs	-	1 and 4 ATU/ml	Promoting the proliferation of HMVECs and activating the VEGF-Notch signaling pathway	Lin et al. (2018)
		7 ATU/ml	Inhibiting the proliferation of HMVECs and the VEGF-Notch signaling pathway	
Random skin flap venous congestion in pigs	Subcutaneous injection	20 and 40 ATU	Inhibiting oxygen free radical damage	Guo-Qian et al. (2012)
**Anti-fibrosis effect**				
UUO-Induced renal interstitial fibrosis in rats	Intravenous injection	10, 20, and 40 IU/kg	Inhibiting the TGF-β1/Smad and NF-κB signaling pathways	Yang et al. (2020)
TGF-β1-induced renal fibrosis in HK-2 cells	-	3 and 10 mg/ml	Inhibiting the JAK/STAT3 signaling pathways	Fan et al. (2018)
Ang II-induced myocardial fibrosis in murine myocardial fibroblasts	-	20, 40, and 80 μg/ml	Inhibiting oxidative stress, regulating fibrosis-related factors, and repressing the ERK1/2 pathway	Yu et al. (2018)
Bleomycin-induced idiopathic pulmonary fibrosis in rats	Subcutaneous injection	25, 50, and 100 ATU/kg	Inhibiting the PI3K/AKT signaling pathways	Zhang et al. (2019)
Bleomycin-induced idiopathic pulmonary fibrosis in rats	Subcutaneous injection	25, 50, and 100 ATU/kg	Inhibiting the mRNA and protein expression levels of PAI-1	Li et al. (2019)
UUO-Induced renal interstitial fibrosis in rats	Oral administration	10 and 15 mg/kg	Inhibiting the inflammation, regulating the related proteins of fibrosis and ETM, and decreasing the apoptosis of renal tubular epithelial cells	Xie et al. (2020)
TGF-β1-induced renal fibrosis in renal tubular epithelial cells	-	0.5 and 1 mg/ml		
**Anti-diabetic complications effects**				
STZ-induced diabetic kidney disease in rats	Subcutaneously injection	1 ATU	Inhibiting the HIF-1α/VEGF signaling pathway	Pang et al. (2020b)
High glucose-treated HK-2 cells	-	10 mg/ml		
STZ-induced diabetic nephropathy in rats	Subcutaneously injection	5 U	Inhibiting the p38 MAPK/NF-κB signaling pathway	Han J. et al. (2020)
High glucose-treated podocytes	-	5 U/ml		
STZ-induced diabetic nephropathy in rats	Subcutaneous injection	5 U		Pang et al. (2020a)
High glucose-treated glomerular endothelial cells	-	5 U/L		
High glucose-treated DRGn	-	0.25, 0.5, and 1 IU/ml	Up-regulating Nrf-2/HO-1 pathway, inhibiting NF-κB pathway, and decreasing the apoptosis of DRGn cell	Liu et al. (2020)
**Anti-tumor effect**				
EOMA cells	-	4 U/ml	Inhibiting the proliferation and promoting the apoptosis	Yang et al. (2015)
human LN229 cells	-	*IC*_*50*_ = 30 mM	Inhibiting the ERK/MAPK signaling pathway, promoting the apoptosis, and increasing cell cycle arrest at the G1 phase	Zhao (2015)
human U251 cells	-	*IC*_*50*_ = 15 mM		
Tumor-bearing H_22_ mice	Oral administration	1 and 2 g/kg	Inhibiting the HGF/c-Met signaling pathway	Yao et al. (2011)
HepG2 cells	-	1, 2, 4, and 8 U/ml	Inhibiting the proliferation, migration, and invasion, promoting the apoptosis, and down-regulating the expression of VEGF.	Li et al. (2016)
CNE2 cells	-	5 and 8 ATU/ml	Inhibiting the proliferation, promoting the apoptosis, and up-regulating the expression of p21	Tao et al. (2018)
			Inhibiting the proliferation, blocking cell cycle at G2/M, and promoting the apoptosis	Liu et al. (2016)
Laryngeal carcinoma tumor-bearing mice	Oral administration	1.2 and 2.5 g/kg	Inhibiting the HGF/c-Met signaling pathway	Ma et al. (2017)
Hep-2 human laryngeal cancer cells	-	0.5, 1 and 2 mg/ml	Inhibiting the proliferation, migration, and invasion, promoting the apoptosis, and reducing the expression of VEGF-R and FAK.	Lu et al. (2015)
		25, 50 and 100 μg/ml		
SMMC-7721 cells	-	1 U/ml	Inhibiting the proliferation, promoting the apoptosis, and reversing thrombin induced growth promotion and apoptosis resistance	Wu and Li (2015)
**Anti-hyperuricemia effect**				
Potassium oxonate-induced hyperuricemia in rats	Oral administration	0.2, 0.4, and 0.8 g/kg	Down-regulating the mRNA and protein expression levels of GLUT9 and URAT1	Wu et al. (2020)
Hypoxanthine-induced hyperuricemia in mice		200, 400, and 800 mg/kg	Down-regulating the protein expression level of GLUT9	Liu et al. (2018)
Sodium uric-induced acute gouty inflammation in rats		200 and 800 mg/kg		
**Effect on cerebral hemorrhage**				
Autologous arterial blood injection-induced experimental cerebral hemorrhage in rats	Local administration	100 U	Inhibiting the apoptosis of neurocytes	Yin and Wei (2013)
		15 U	Inhibiting the apoptosis of neurocytes and the JAK2/STAT3 signaling pathway	Li et al. (2017)
**Other effects**				
Bovine gamma-globulin- induced IgAN model in rats	Oral administration	10 mg/kg	Suppressing fibrosis, maintaining the balance of immune system, and inhibiting the IκBα, NF-κB, TNF-α, and VCAM-1 signaling pathways	Deng et al. (2019)
LPS-induced acute lung injury model in rats	Intraperitoneal injection	1, 2, and 3 ATU	Inhibiting the production of PAR-1 and reduced the content of TNF-α and MMP12	Bao et al. (2012)
Isoproterenol-induced myocardial infraction in rats	Oral administration	15 and 30 mg/kg	Activating the Nrf2 signaling pathway through disrupting Keap1-Nrf2 complex	Zhang et al. (2020)
Hypoxia-Reoxygenation model in H9C2 cells	-	5, 10, and 20 μM		

Although hirudin has been successfully used for thrombosis therapy in clinic, its short half-life and the bleeding risk remain limiting factors, further researches may focus on the structural modifications. Perhaps, fusing recognition peptides identified from FXIa and FXa with these drugs might be a promising method to reduce bleeding risk. In addition, the studies associated with the derivatives and preparations of hirudin are only at the preliminary stage and further studies should be conducted to reveal the actions, toxicity and potential mechanisms.

In short, hirudin has a wide range of pharmacological activities and enormous potential for clinic application, which is worthy of more in-depth and comprehensive study.

## Glossary

Ang II: angiotensin II
a-SMA: α-smooth muscle actin
ASST: arylsulfotransferase
BSA: bovine serum albumin
BUN: blood urea nitrogen
CK-MB: Creatine Kinase Isoenzyme-MB
COL-I: collagen-I
DE3: Escherichia coli BL21
DKD: diabetic kidney disease
DN: diabetic nephropathy
DRGn: dorsal root ganglion neurons
DTIP: direct thrombin inhibitor peptide
ECM: extracellular matrix
EMT: epithelial-mesenchymal transition
ERK: extracellular regulated protein kinases
ERK1/2: extracellular signal-regulated kinase1/2
FAK: focal adhesion kinase
FN: fibronectin
GLUT9: glucose transporter 9
GPCRs: G protein–coupled receptors
hAvHA: Annexin V–hirudin 3-ABD
HCC: hepatocellular carcinoma
HG: high glucose
HIF-1: hypoxia-inducible factor 1
HMVECs: attenuate human microvascular endothelial cells
HO-1: heme oxygence-1
HUA: Hyperuricemia
ICAM-1: intercellular adhesion molecule-1
ICH: Intracerebral hemorrhage
IgAN: IgA nephropathy
IPF: idiopathic pulmonary fibrosis
JAK/STAT: Janus kinase/signal transduction and activator of transcription
LDH: lactate dehydrogenase
MAPK: mitogen-activated protein kinase
MCP-1: Monocyte chemoattractant protein-1
MDA: malonaldehyde
MMP-2: matrix metalloproteinase-2
MVD: vascular density value
Nrf-2: nuclear factor erythroid 2-related factor-2
PAI-1: plasminogen activator inhibitor-1
PARs: Proteinase-activated receptors
OAT: organic anion transporters
RGD: Arg-Gly-Asp
rH: recombinant hirudin
RIF: renal interstitial fibrosis
ROS: reactive oxygen species
SBI: secondary brain injury
SOD: superoxide dismutase
STZ: streptozotocin
TCM: traditional Chinese medicine
TGF-β1: transforming growth factor β1
T RI: type I receptor
T RII: type II receptor
TSP-1: thrombospondin-1
TIMP-2: metalloproteinases-2
UUO: unilateral ureteral obstruction
uPA/tPA: urokinase/tissue type plasminogen activator
URAT1: novel urate transporter 1
VEGF: vascular endothelial growth factor
